# Bioinformatics and Machine Learning Methods to Identify FN1 as a Novel Biomarker of Aortic Valve Calcification

**DOI:** 10.3389/fcvm.2022.832591

**Published:** 2022-02-28

**Authors:** Tao Xiong, Shen Han, Lei Pu, Tian-Chen Zhang, Xu Zhan, Tao Fu, Ying-Hai Dai, Ya-Xiong Li

**Affiliations:** ^1^Cardiovascular Surgery, Yan'an Affiliated Hospital of Kunming Medical University, Kunming Medical University, Kunming, China; ^2^Key Laboratory of Cardiovascular Disease of Yunnan Province, Yan'an Affiliated Hospital of Kunming Medical University, Kunming Medical University, Kunming, China; ^3^Department of Thoracic and Cardiovascular Surgery, The First Affiliated Hospital of Chongqing Medical University, Chongqing, China

**Keywords:** aortic valve calcification, immune infiltration, diagnostic, biomarker, CIBERSORT, small molecule drugs

## Abstract

**Aim:**

The purpose of this study was to identify potential diagnostic markers for aortic valve calcification (AVC) and to investigate the function of immune cell infiltration in this disease.

**Methods:**

The AVC data sets were obtained from the Gene Expression Omnibus. The identification of differentially expressed genes (DEGs) and the performance of functional correlation analysis were carried out using the R software. To explore hub genes related to AVC, a protein–protein interaction network was created. Diagnostic markers for AVC were then screened and verified using the least absolute shrinkage and selection operator, logistic regression, support vector machine-recursive feature elimination algorithms, and hub genes. The infiltration of immune cells into AVC tissues was evaluated using CIBERSORT, and the correlation between diagnostic markers and infiltrating immune cells was analyzed. Finally, the Connectivity Map database was used to forecast the candidate small molecule drugs that might be used as prospective medications to treat AVC.

**Results:**

A total of 337 DEGs were screened. The DEGs that were discovered were mostly related with atherosclerosis and arteriosclerotic cardiovascular disease, according to the analyses. Gene sets involved in the chemokine signaling pathway and cytokine–cytokine receptor interaction were differently active in AVC compared with control. As the diagnostic marker for AVC, fibronectin 1 (FN1) (area the curve = 0.958) was discovered. Immune cell infiltration analysis revealed that the AVC process may be mediated by naïve B cells, memory B cells, plasma cells, activated natural killer cells, monocytes, and macrophages M0. Additionally, FN1 expression was associated with memory B cells, M0 macrophages, activated mast cells, resting mast cells, monocytes, and activated natural killer cells. AVC may be reversed with the use of yohimbic acid, the most promising small molecule discovered so far.

**Conclusion:**

FN1 can be used as a diagnostic marker for AVC. It has been shown that immune cell infiltration is important in the onset and progression of AVC, which may benefit in the improvement of AVC diagnosis and treatment.

## Introduction

Aortic valve calcification (AVC) is the most frequent kind of valvular disease in the world, and is the most common cause of aortic stenosis (1, 2). There are between 2 and 7% of the population aged 65 and older who are affected by this disease (3). As the condition worsens over time, more and more fibrous calcification and pathological thickening of the aortic valve lead to a reduction in the mobility of the aortic valve leaflets, and ultimately lead to serious obstruction of the heart outflow (4). The disease burden of AVC is expected to double over the next 50 years, posing a serious threat to public health in an aging world (4). Although the therapies used to treat aortic valve stenosis have made progress, including valve replacement and interventional therapy, the exact biological process of AVC is still mystery.

After statins failed to slow the progression of AVC, angiotensin-converting enzyme inhibitors also failed, and there is still no progress in the effectiveness of drug treatments (5–8). To date, early surgery is the only effective treatment option for improving clinical outcomes in patients with AVC (9). However, surgery is related to high expenses, an inevitability of mortality, as well as perioperative and long-term morbidity, such as those related with anticoagulant medication and the necessity for reoperation due to prosthetic valve failure. As a result, an urgent medical need was recognized for a comprehensive understanding of fundamental processes of AVC as well as for the development of new treatment targets to slow the advancement of the disease's evolution. In order to diagnose and cure early, biomarkers and pathways should be identified. This is essential for early recognition, prevention, and accurate therapy.

Histopathological analysis reveals that the beginning phase of AVC is an aggressive inflammation reaction, similar to that of atherosclerotic lesions, consisting of processes ranging from lipid deposition, infiltration of inflammatory cells such as macrophages and T cells, and finally leading to the destruction of the basement membrane (10). The final stage of AVC is typically characterized by heterotopic ossification, which includes mature lamellar bone formation and active bone remodeling (11). According to several studies, signal transduction channels that are associated with the progression of aortic calcification are composed of various growth factors, cytokines, and tumor necrosis factors (12–14). Current knowledge of the mechanisms underlying AVC progression, including molecular actions, cellular functions and biomechanics, has been established. The most important of these are the morphology of the mitral aortic valve, disturbances in endocrine regulation, valve osteogenesis, dysregulation of mineral metabolism, and lack of signaling by osteoclasts (15).

In this study, we used CIBERSORT for the first time to analyze the expression matrix of AVC and normal tissue samples, and calculated the proportions of their immune cells. Moreover, we investigated the association between the biomarkers discovered and the infiltrating immune cells, providing the groundwork for future study in this area. Most importantly, we have well screened potential AVC targeted small molecule drugs, by using the connection map (CMap) database.

## Materials and Methods

### Data Download and Processing

The GSE12644 (16) and GSE51472 (17) datasets were obtained from http://www.ncbi.nlm.nih.gov/geo/ (18), which the GPL570 platform of the 84 Affymetrix Human Genome U133 Plus 2.0 Aray served as the foundation. The GSE12644 dataset contained 10 AVC and 10 controls that were collected from the aortic valve, and the GSE51472 dataset contained 10 AVC and 5 controls that were collected from the aortic valve. The probes in each dataset were translated to gene symbols based on the probe annotation files that were provided by the researchers. As several probes correspond to the same gene symbol, we use the average value of the probes to represent the level of expression of that gene in the tissue. In order to conduct additional integration research, the two datasets were pooled into a metadata cohort. This was done since GSE12644 and GSE51472 both share a similar platform and are beneficial for merging data. Before performing normalization, the expression values from both datasets were log2 transformed. ComBat outperforms other tools in a systematic evaluation. As a result, we choose ComBat in order to eliminate the batch effect between the two datasets (19). We performed principal component analysis (PCA) to determine if the batch effect had been eliminated. Additionally, we identified an aortic valve disease data set (GSE83453) (20) from the GEO database to serve as a validation cohort, consisting of nine AVC samples and eight control samples, which was performed using the Illumina HumanHT-12 v4.0 Gene Expression BeadChip (Laval University, Quebec, Canada; anthor:Yohan Bossé).

### Identification of DEGs

The “limma” package (21) was used to screen for DEGs, and heat and volcano maps of DEGs were created by the “ggplot2” package (22) to visualize their differential expression. With the P value < 0.05 and |log2FC| > 0.585, DEGs were deemed statistically significant in this study.

### Functional Correlation Analysis

We performed Gene Ontology (GO), Kyoto Encyclopedia of Genes and Genomes (KEGG) and Disease Ontology (DO) enrichment analyses of DEGs using the “clusterProfiler” package (23). Gene set enrichment analysis (GSEA) of the gene expression matrix was performed using the “clusterProfiler” package, with the reference gene sets “c5.go.v7.4.symbols.gmt” and “c2.cp.kegg.v7.0.symbols.gmt” being used. *q*-value < 0.05 and adjusted *P-*value < 0.05 were considered significantly enriched.

### Hub Genes Screening

The Search Tool for the Retrieval of Interacting Genes (STRING) (24) database was used to analyze functional protein association networks. The screened DEGs were submitted to the STRING database. All protein–protein interaction (PPI) pairs with a combined score of >0.7 were extracted. To maintain the overall network's stability, high-degree nodes seemed to be essential. We use Cytoscape (v3.6.1) plug-in cytoHubba to calculate the degree of all nodes (25). In this study, the top 10 genes with the greatest degree rank are identified key genes.

### Verification of Diagnostic Markers

To screen diagnostic markers for AVC, the least absolute shrinkage and selection operator (LASSO) logistic regression (26) as well as support vector machine-recursive feature elimination (SVM-RFE) (27) were used by us. After quality control, the expression matrices of the GSE12644 and GSE51472 datasets were combined to create an independent dataset, and the combined diagnosing efficacy of the gathered diagnostic markers was determined using this dataset. The LASSO algorithm was used in conjunction with the “glmnet” package (28). SVM-RFE is a support vector machine-based machine learning technique, which is used to exclude SVM-generated eigenvectors in order to select the optimal variables. The “e1071” package established an SVM module to further characterize the diagnostic utility of the biomarkers in AVC (29). We integrated the hub genes with the PPI network, LASSO and SVM-RFE algorithms to conduct a more in-depth study. *P* < 0.05 was regarded statistically significant on a two-sided basis.

### Evaluation of Immune Cell Infiltration

In CIBERSORT (https://cibersortx.stanford.edu/), we filtered out samples with a *P* < 0.05 and acquired the matrix about immune cell infiltration as a result of submitting the gene expression matrix information. The “ggplot2” software was then used to conduct PCA analysis on the immune cell infiltration matrix input, resulting in the creation of a two-dimensional PCA map. The “corrplot” software is responsible for plotting correlative data. Correlation heatmaps were created using the “corrplot” package (30) in order to show the relationship between 22 distinct kinds of infiltration immune cells. It was necessary to build violin diagrams to display the discrepancies in immune cell infiltration, which was accomplished using the “ggplot2” package.

### Immune Cells and Diagnostic Markers

The relationship between the amounts of infiltrating immune cells and the levels of the discovered gene biomarkers was investigated using Spearman's rank correlation test in the R software. The chart method provided by the “ggplot2” package was used to display the generated correlations.

### Identification of Small Molecules

CMap (http://www.broadinstitute.org/cmap/) is a repository of databases containing thousands of gene transcription profiles, obtained from cultured mammalian cells exposed to active small molecule drugs. It was searched to identify small molecule therapeutic candidates with the AVC gene signature. DEGs were classified into two groups: up-regulated and down-regulated groups. The similarity was quantified using enrichment scores ranging from −1 to +1. A positive connecting value (near to +1) suggests that a small compound may trigger the expression of the AVC gene, while a negative connecting value (near to −1) implies that a compound can mimic the condition of normal cells.

## Results

Supplementary File 1 lists data matrix information of the training datasets (GSE12644 and GSE51472) and validation dataset (GSE83453).

### Data Preprocessing and DEG Screening

**Figure 1:**
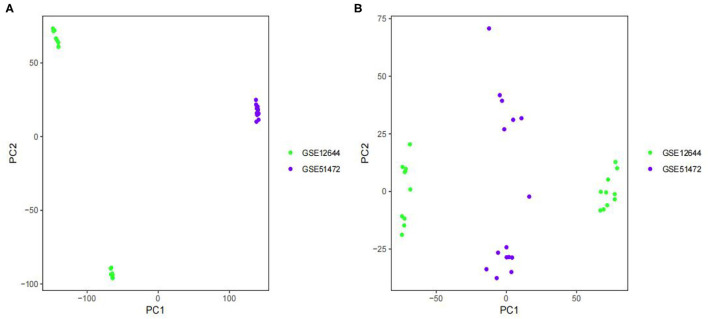
Principal component analysis (PCA) analysis of gene expression datasets. The scatter plots' points depict samples based on the top two principal components (PC1 and PC2) of gene expression profiles without and with batch effect removal. **(A)** PCA cluster plot of GSE12644 and GSE51472 before sample correction and remove batch effect. **(B)** PCA cluster plot of GSE12644 and GSE51472 after sample correction and remove batch effect. The colors denote samples from two distinct datasets, respectively. Each dot represents a sample; green represents a sample from GSE12644; purple represents a sample from GSE51472.

**Figure 2:**
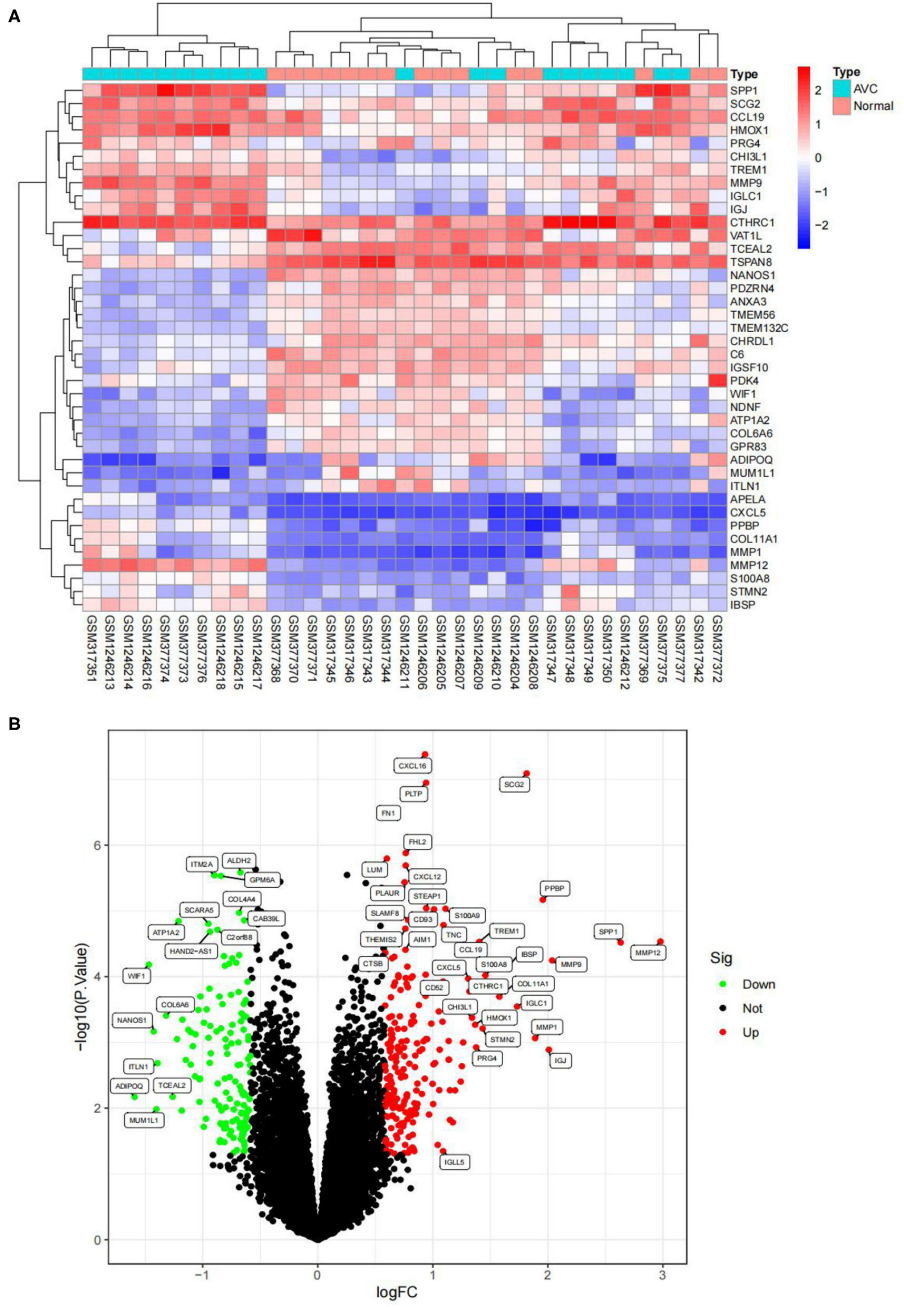
Visualizing the results of differential genes. **(A)** Clustering heatmap of the genes exhibiting significantly differential expression AVC vs. normal group. Statistically significant DEGs were defined as |log2Foldchange| > 0.585 and *P*-value < 0.05. DEG, differentially expressed gene; AVC, Aortic valve calcification group. Cyan represents AVC groups; red-orange represents normal groups. **(B)** Volcano map of DEGs; red represents up-regulated differential genes, black represents no significant difference genes, and green represents down-regulated differential genes.

The batch effect between GSE12644 and GSE51472 was evaluated and visualized using a PCA cluster diagram. A batch effect existed between them (Figure 1A), and the inter-batch variation had been eliminated. After normalization and processing, two two-dimensional PCA cluster diagrams were used to display the combined gene expression matrix before normalization (Supplementary File 2) and after normalization (Supplementary File 3), respectively (Figure 1B). After normalization, the clustering of the two sample groups was more evident, indicating a reliable sample source. Following data preparing, we used R to identify 337 DEGs from the normalized data, as described by the heat map as well as volcano map (Figures 2A,B). Compared with normal samples, 203 of these DEGs were upregulated and 134 were downregulated in AVC. The top 10 upregulated and downregulated genes are summarized in Table 1.

**Table 1:** The top 10 up- and down-regulated DEGs in AVC and normal sample.

**Gene symbol**	**Fold-change**	***P*-value**
**Top 10 up-regulated DEGs**		
MMP12	2.979149817	2.92E-05
SPP1	2.632830567	3.03E-05
MMP9	2.037460333	5.66E-05
IGJ	2.008886783	0.001292002
PPBP	1.956678033	6.77E-06
MMP1	1.889498133	0.000862519
SCG2	1.815960233	8.12E-08
IGLC1	1.732297767	0.000284768
IBSP	1.645398033	7.50E-05
COL11A1	1.577731517	0.000201496
**Top 10 down-regulated DEGs**		
ADIPOQ	−1.5926205	0.006759383
WIF1	−1.46850865	6.57E-05
NANOS1	−1.426355333	0.000681789
MUM1L1	−1.403391017	0.010340331
ITLN1	−1.393083817	0.002074118
COL6A6	−1.318182983	0.000392129
TCEAL2	−1.260862617	0.006721387
TSPAN8	−1.223407683	0.000891201
ATP1A2	−1.209701467	1.43E-05
PDZRN4	−1.1820793	0.010880592

### Functional Correlation Analysis

**Figure 3:**
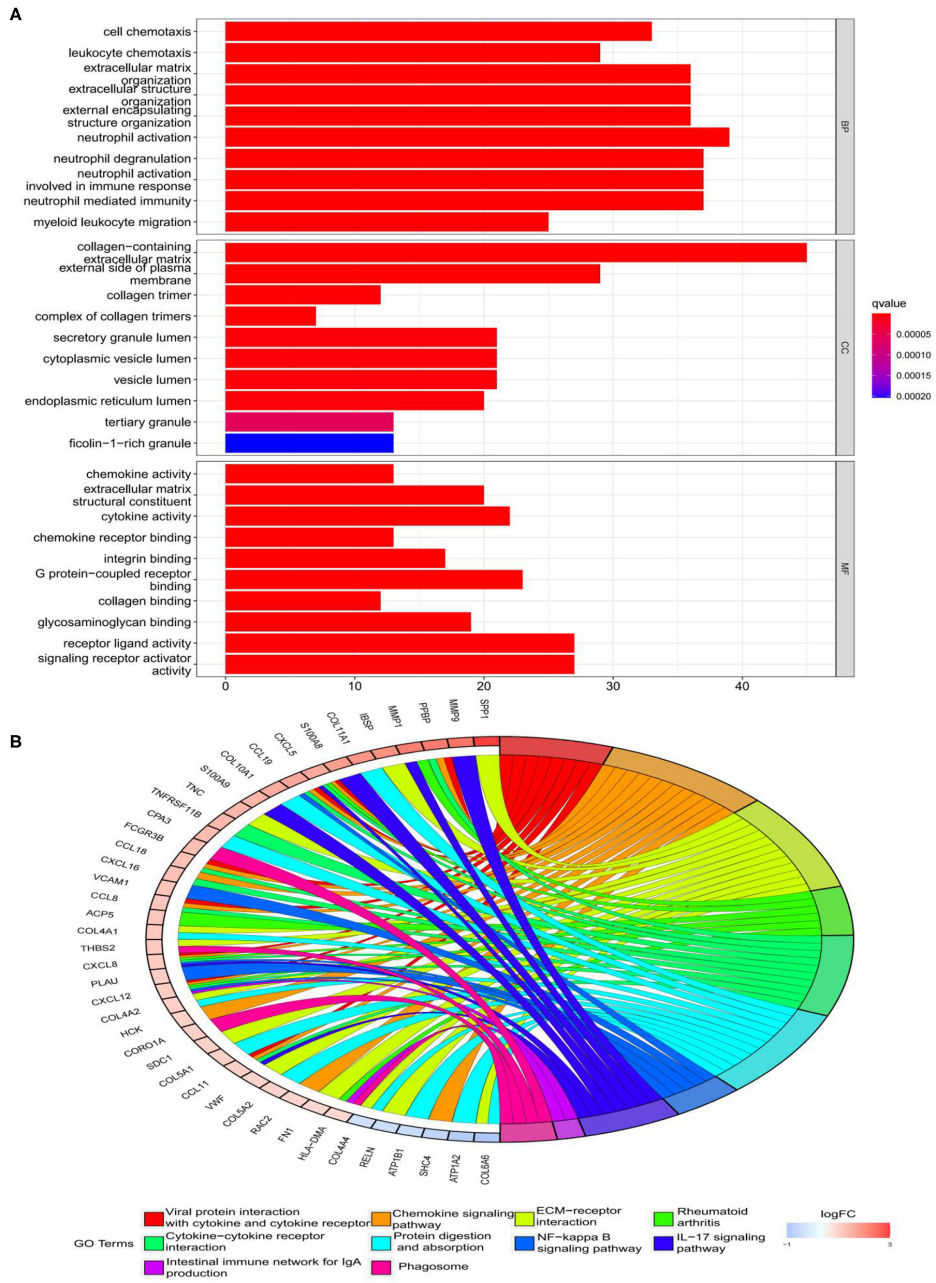
Functional enrichment analyses of DEGs. **(A)** Gene Ontology (GO) enrichment analyses of DEGs. The x-axis shows the number of genes enriched on the terms, and the y-axis shows the pathway terms. The q-value of each term is colored according to the legend. BP, biological process; CC, cellular component; MF, molecular function; **(B)** Kyoto Encyclopedia of Genes and Genomes (KEGG) enrichment analyses of DEGs. Select the first 30 DEGs to connect to the enriched pathway terms. The q-value of each term is colored according to the legend. The different colors represent different pathway terms.

**Figure 4:**
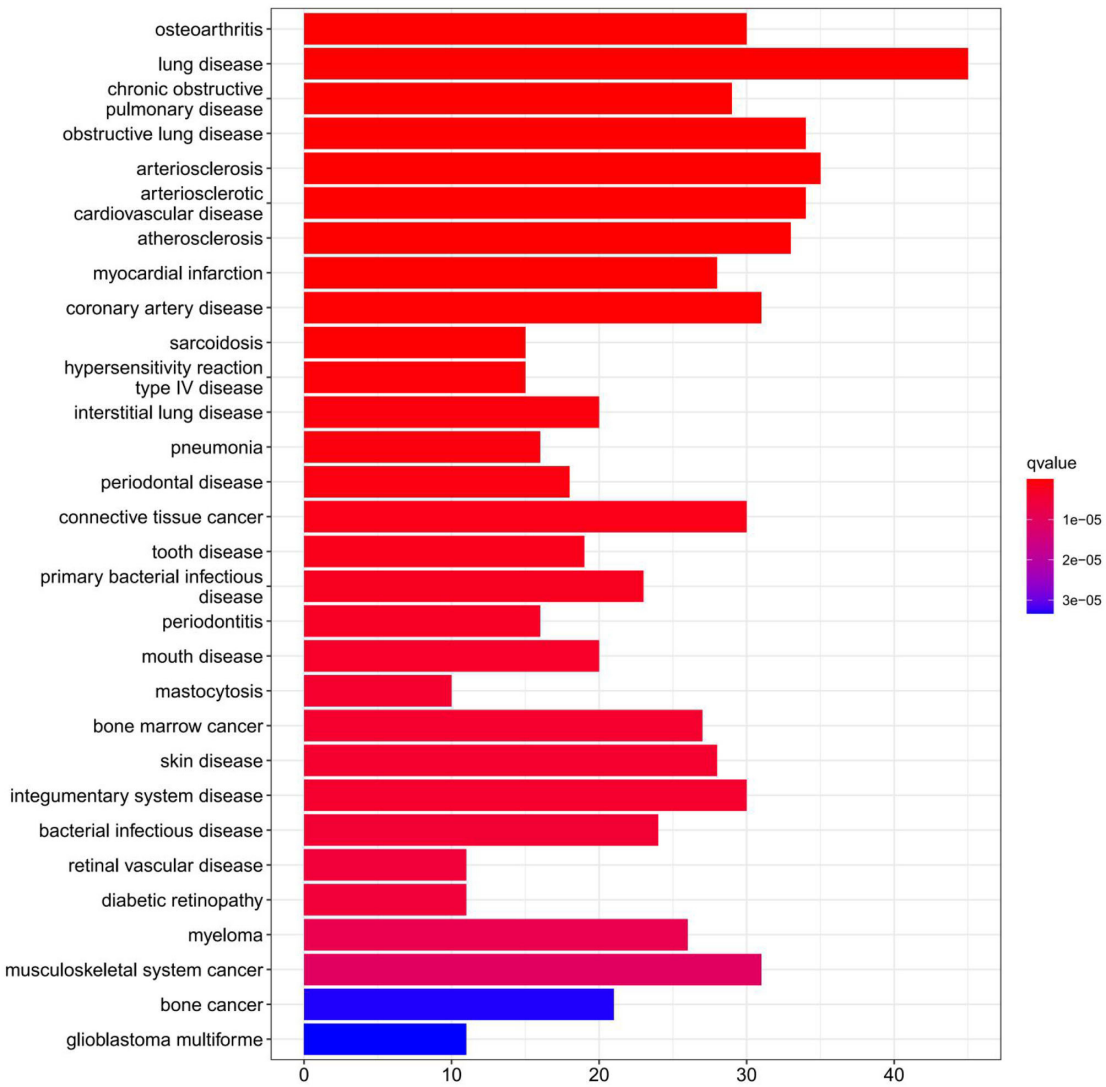
Disease Ontology (DO) enrichment analysis was performed on DEGs and the top 30 terms were selected for visualization. The x-axis shows the number of genes enriched on the terms and the y-axis shows the pathway terms. The q-value of each term is colored according to the legend.

**Figure 5:**
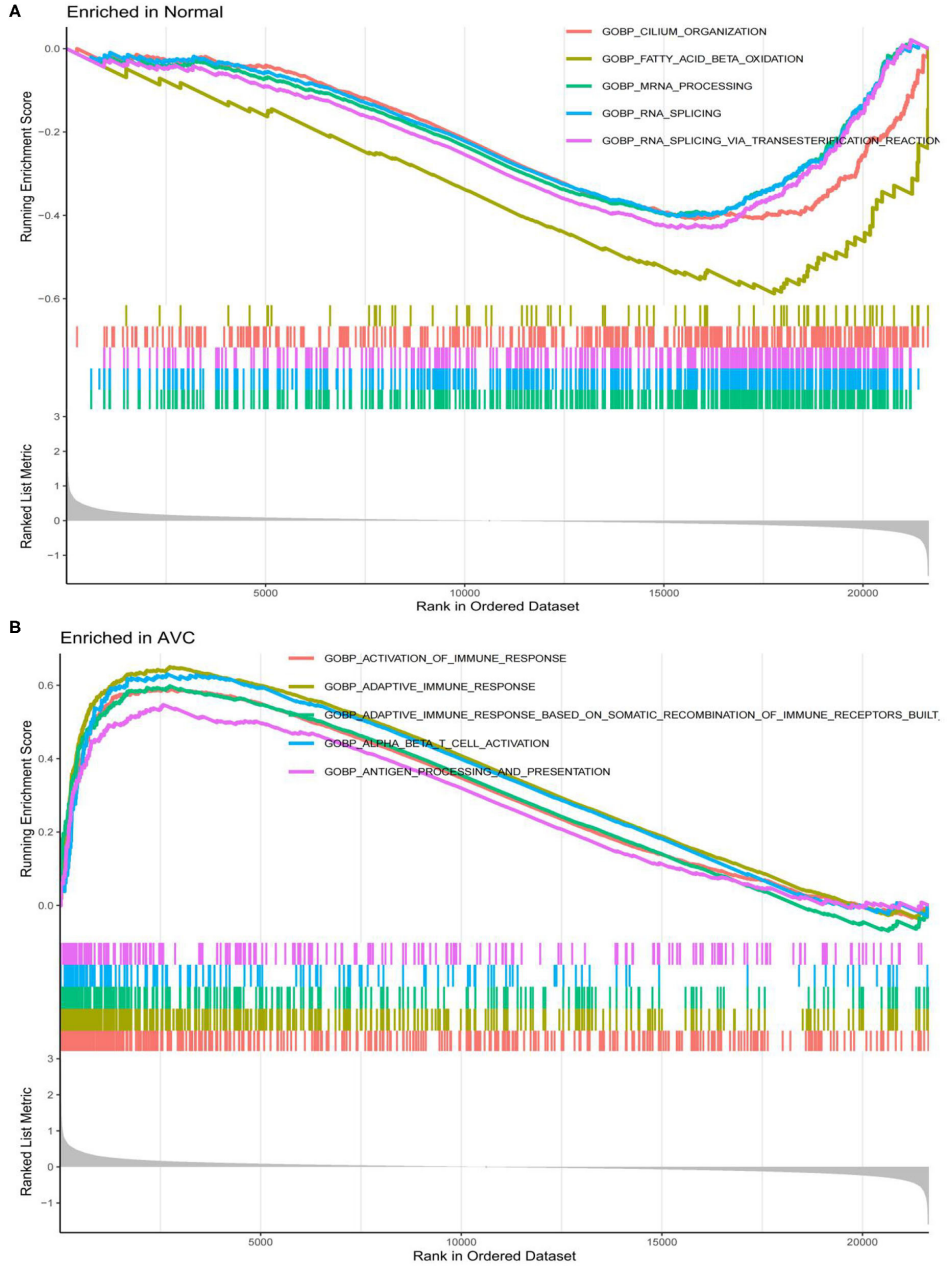
LogFC values were calculated for all genes, and Gene set enrichment analysis (GSEA) analysis was performed based on logFC using c2.cp.kegg.v7.4.symbols.gmt and c5.go.v7.4.symbols.gmt in the normal and AVC groups using. **(A)** Analysis of the GO pathway terms for all genes enriched in the normal group using GSEA. **(B)** Analysis of the GO pathway terms for all genes enriched in the AVC group using GSEA.

**Figure 6:**
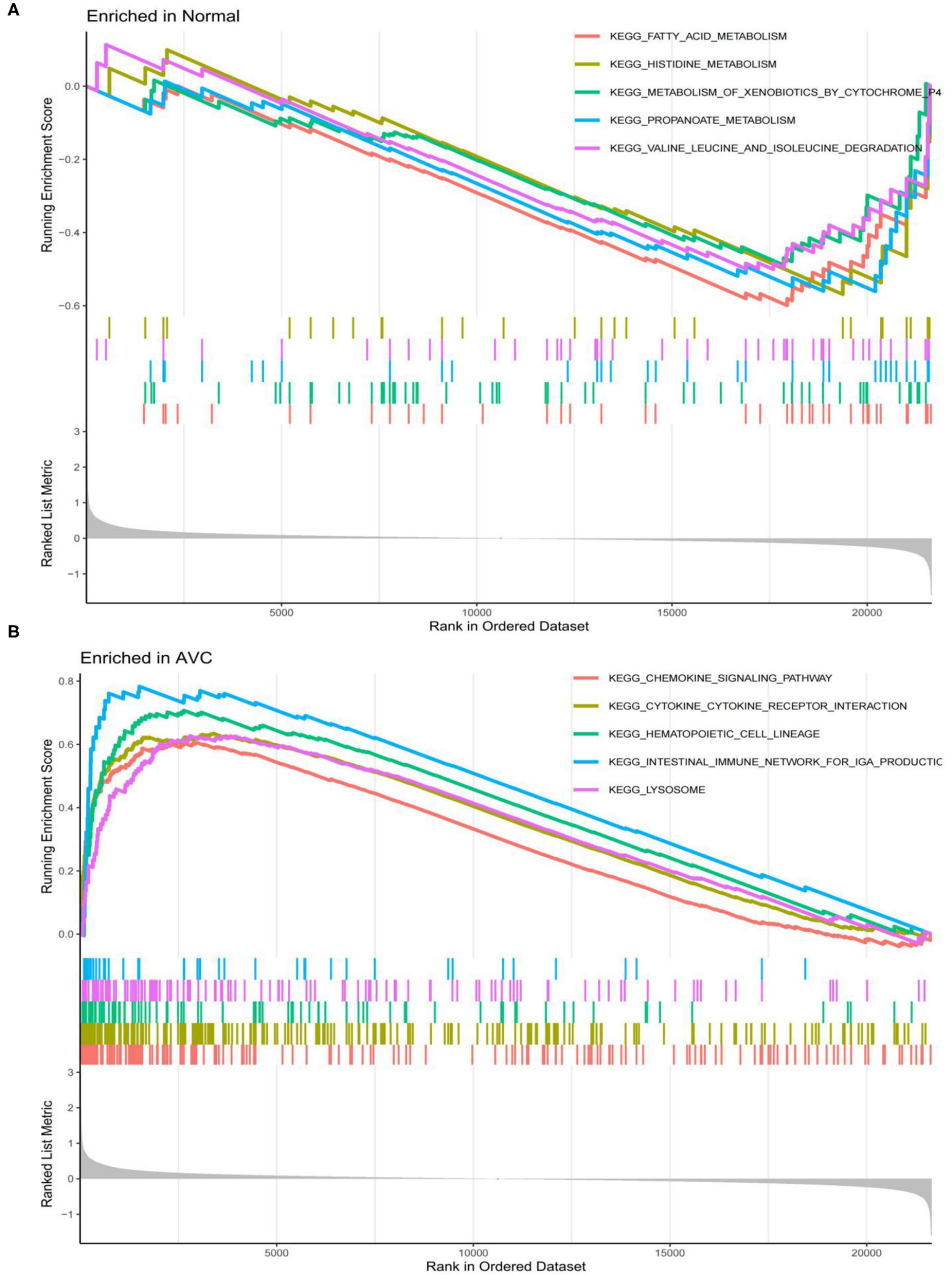
Analysis of the top 4 Kyoto Encyclopedia of Genes and Genomes (KEGG) pathway terms for all genes enriched in the normal and AVC groups using Gene set enrichment analysis (GSEA). **(A)** Analysis of the KEGG pathway terms for all genes enriched in the normal group using GSEA. **(B)** Analysis of the KEGG pathway terms for all genes enriched in the AVC group using GSEA.

The findings of the GO analysis were separated into three subcategories, which were biological process, cell component, and molecular function, respectively (Figure 3A, Supplementary File 4). Cell chemotaxis, leukocyte chemotaxis, extracellular matrix organization, extracellular structure organization, external encapsulating structure organization, neutrophil activation, neutrophil degranulation, neutrophil activation involved in immune response, neutrophil-mediated immunity, and myeloid leukocyte migration were all observed during the biological process. The DEGs were enriched in cell components including the extracellular matrix containing collagen, the external side of the plasma membrane, collagen trimer, complex of collagen trimers, secretory granule lumen, cytoplasmic vesicle lumen, vesicle lumen, endoplasmic reticulum lumen, tertiary granule, and ficolin-1-rich granule. The DEGs were enriched in chemokine activity, extracellular matrix structural constituent, cytokine activity, chemokine receptor binding, and integrin binding for molecular function. Otherwise, the KEGG pathway was enriched for viral protein interaction with cytokine and cytokine receptor, chemokine signaling pathway, extracellular matrix-receptor interaction, rheumatoid arthritis, and cytokine–cytokine receptor interaction (Figure 3B, Supplementary File 4). The findings of the DO study are depicted in Figure 4 and Supplementary File 4. The most common diseases enhanced by DEGs were osteoarthritis, lung disease, chronic obstructive pulmonary disease, obstructive lung disease, arteriosclerosis, arteriosclerotic cardiovascular disease, atherosclerosis, myocardial infarction, and coronary artery disease. GSEA results indicated that in normal samples, GO biological processes primarily involved fatty acid beta oxidation, mRNA processing, RNA splicing, and RNA splicing *via* transesterification reaction (Figure 5A, Supplementary File 5). In disease samples, GO biological processes primarily involved inactivation of immune response and adaptive immune response based on somatic recombination of immune receptors built (Figure 5B, Supplementary File 5). The KEGG enrichment analysis in the normal group (Figure 6A, Supplementary File 5) revealed significant enrichment in fatty acid metabolism, histidine metabolism, xenobiotic metabolism via cytochrome p4, propanoate metabolism, and valine leucine and isoleucine degradation. In the disease group, significantly enriched in the KEGG pathway (Figure 6B, Supplementary File 5) including chemokine signaling pathway, cytokine-cytokine receptor interaction, and hematopoietic cells. The aforementioned results indicate that that the immune response significantly affects the process of AVC.

### Hub Genes Screening

**Figure 7:**
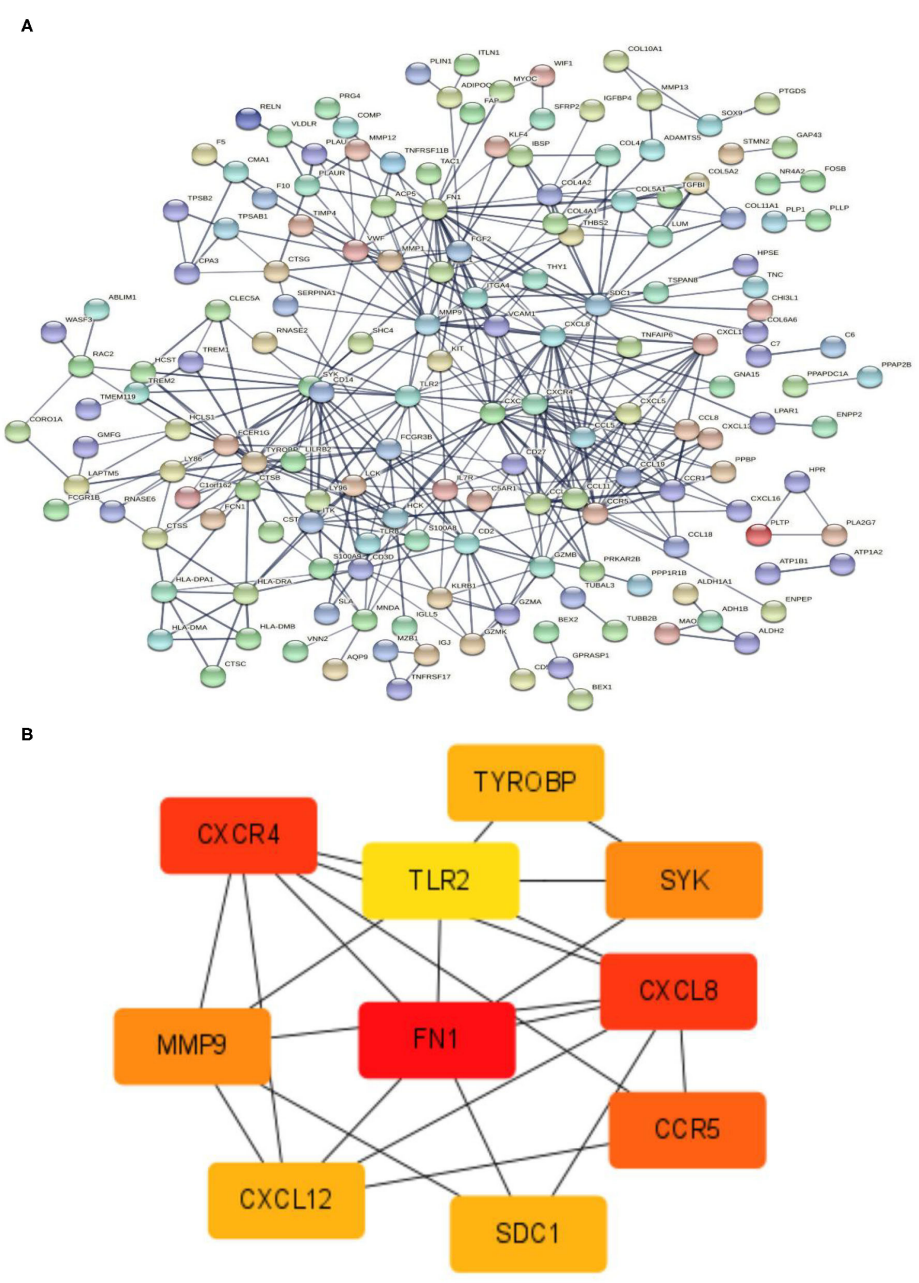
PPIN and hub gene identification. **(A)** PPIN was constructed by all the 337 DEGs using STRING database. The protein-protein interactions are represented by linkages. The number of nodes: 320; the number of edges: 420; the average node degree: 2.62; the PPI enrichment *p*-value < 1.0e-16. **(B)** The top 10 hub genes in the PPIN were screened by Cytoscape (v3.6.1) plugin cytoHubba based on their connectivity degree. The 10 identified hub genes such as FN1, CXCL8, CXCR4, CCR5, MMP9, SYK, TYROBP, CXCL12, SDC1, and TLR2 are displayed from red (high degree value) to yellow (low degree value). PPIN, protein-protein interaction network; DEG, differentially expressed gene; STRING, search tool for the retrieval of interacting genes.

STRING tools were used to predict the protein-protein interactions of the 337 DEGs. The protein-protein interaction network contained 165 nodes and 420 edges (Figure 7A). The top 10 key genes are identified by the degree of connectivity in the protein-protein interaction network. With a connection degree of 28, fibronectin 1 (FN1) was the most prominent gene, followed by C-X-C motif chemokine ligand 8 (CXCL8) (degree = 23), C-X-C motif chemokine receptor 4 (CXCR4) (degree = 23), C-C motif chemokine receptor 5 (CCR5) (degree = 20), matrix metallopeptidase 9 (MMP9) (degree = 19), spleen-associated tyrosine kinase (SYK) (degree = 19), transmembrane immune signaling adaptor TYROBP (TYROBP) (degree = 18), C-X-C motif chemokine ligand 12 (CXCL12) (degree = 18), syndecan 1 (SDC1) (degree = 18), and toll-like receptor 2 (TLR2) (degree = 17). All of the key genes listed above are upregulated. Additionally, the PPIN of the 10 discovered hub genes were created, indicating a substantial interaction between them (Figure 7B).

### Verification of Diagnostic Markers

**Figure 8:**
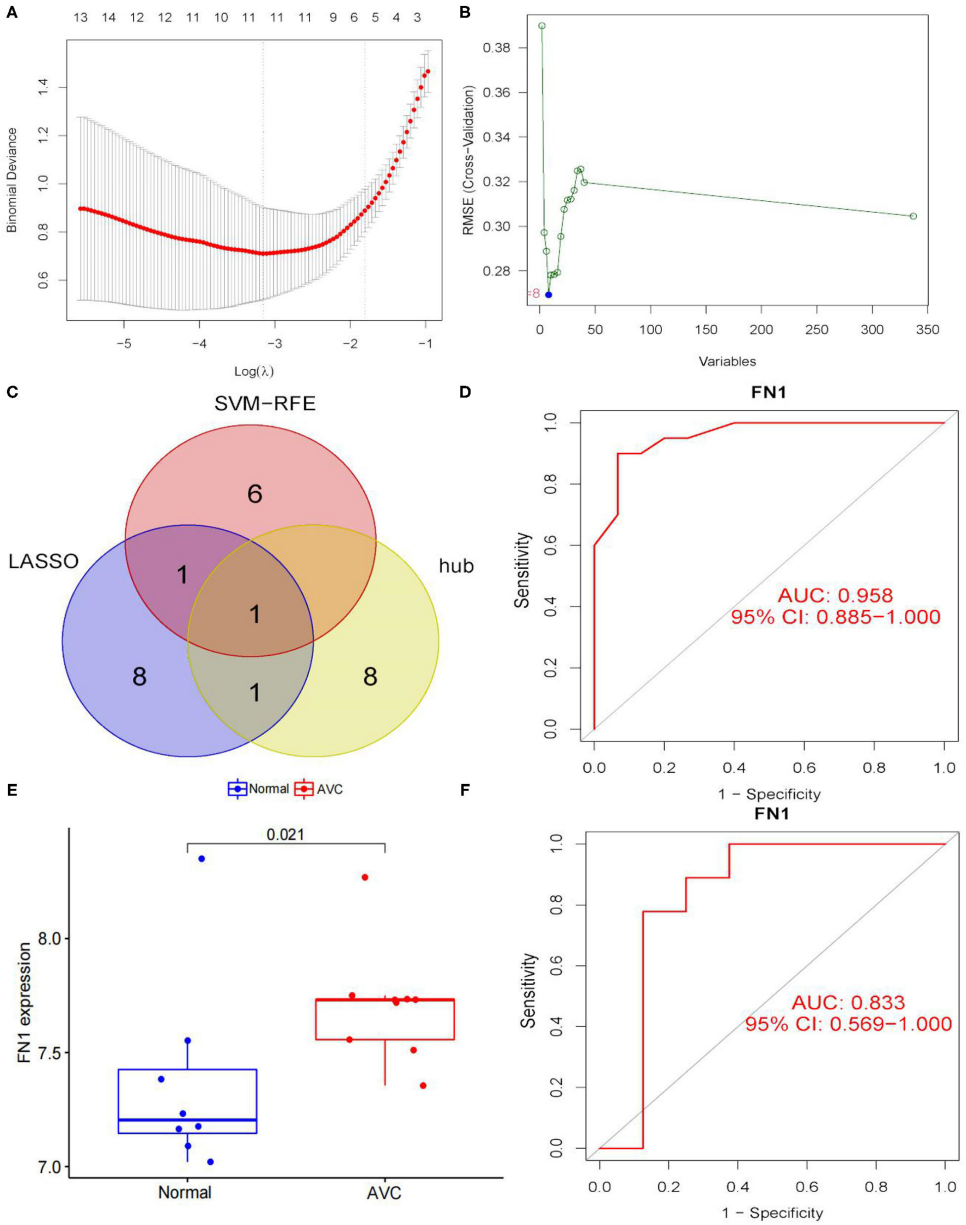
Screening and verification of diagnostic markers. **(A)** Tuning feature selection in the least absolute shrinkage and selection operator (LASSO) model. The DEGs were narrowed down using the LASSO regression algorithm, resulting in the identification of 11 variables as diagnostic biomarkers for AVC. The ordinate is the value of the coefficient, the lower abscissa is log(λ), and the upper abscissa is the number of non-zero coefficients in the model at this time. **(B)** A plot of biomarkers selection via support vector machine-recursive feature elimination (SVM-RFE) algorithm. A subset of five features among the DEGs was determined using the SVM-RFE algorithm. **(C)** Venn diagram demonstrating one diagnostic marker shared by the least absolute shrinkage and selection operator and SVM-RFE algorithms and hub genes. **(D)** The diagnostic performance of the calculated based on the FN1 expression in AVC diagnosis in training data set. **(E)** FN1 mRNA expression in AVC compared to normal groups in the validation set. **(F)** The diagnostic performance of the calculated based on the FN1 expression in AVC diagnosis in test data set. The distinction was considered good when the AUC value was between 0.8 and 0.9, and exceptional when the AUC value was > 0.9. ROC, receiver operating characteristic; AUC, area under the ROC curve.

To use the LASSO logistic regression approach, we were able to recognize 11 genes from DEGs (Figure 8A). The SVM-RFE technique is used to determine eight genes from DEGs (Figure 8B). By combining the genes identified by the two techniques and the hub gene, a diagnostic-related gene was generated (Figure 8C). FN1 was matched to a variable and had a diagnostic efficiency of 0.958 in the training set (Figure 8D). To further evaluate the diagnostic efficacy of FN1, we used the GSE83453 dataset as the validation set (Figure 8E). FN1 achieved a high level (area the curve = 0.833) (Figure 8F), showing that FN1 had a high diagnostic value.

### Results of Immune Cell Infiltration

**Figure 9:**
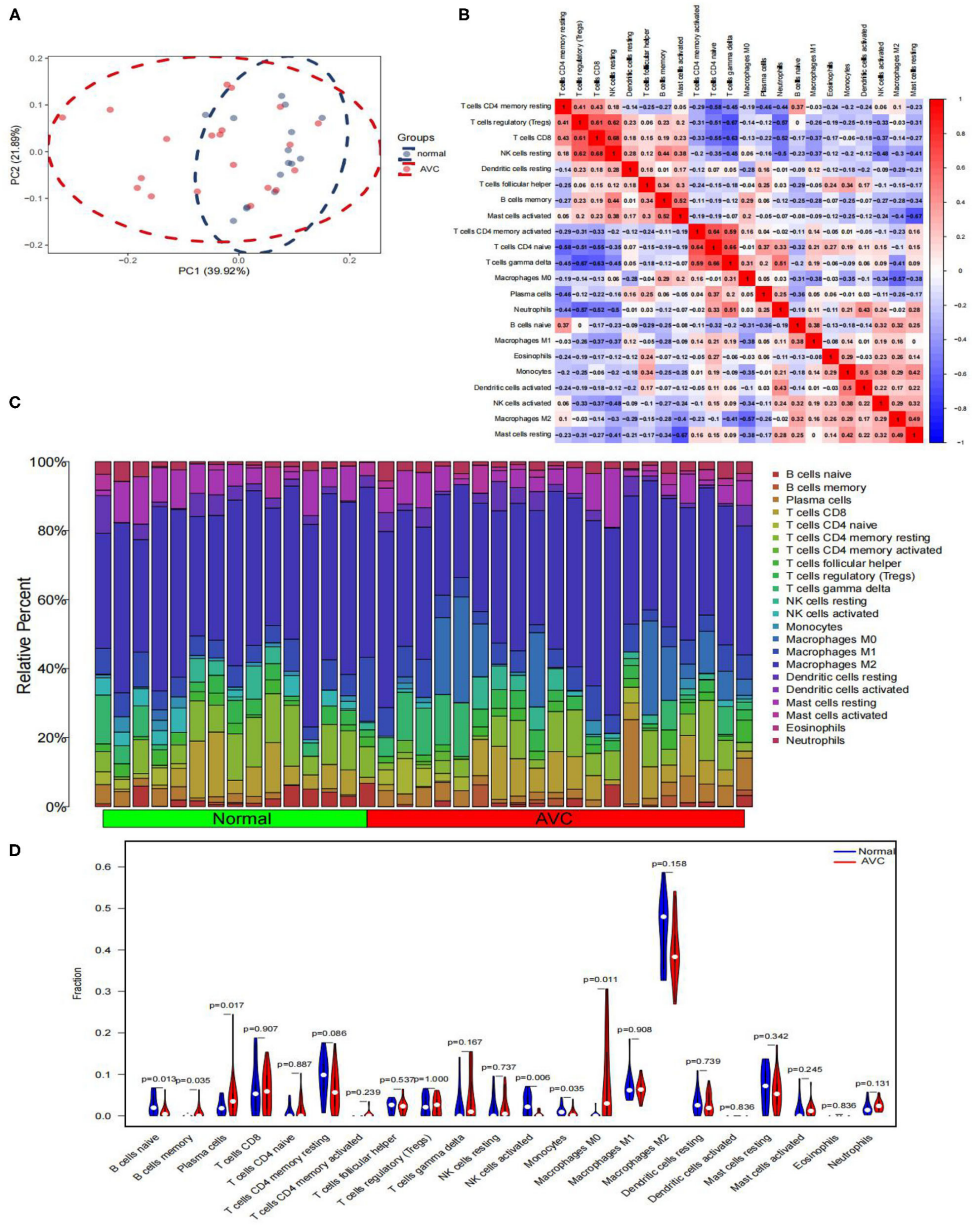
Comparing the composition of immune cell infiltration in the normal and AVC samples by using the combined data matrix of GSE12644 and GSE51472 and visualized the results. **(A)** PCA cluster plot of immune cell infiltration between normal and AVC groups. **(B)** Correlation heat map of 22 types of immune cells. The size of the colored squares represents the strength of the correlation; red represents a positive correlation, blue represents a negative correlation. The redder the color, the stronger the correlation. **(C)** The heat map of the 22 subpopulations of immune cells. **(D)** Violin diagram of the proportion of 22 types of immune cells. (The normal controls group was marked as blue color and AVC group was marked as red color. *P*-values < 0.05 were considered as statistically significant.)

GSE12644 and GSE51472 merged data matrices were analyzed using CIBERSORT and the results are described in Supplementary File 6. In order to examine the uniformity of biological repetition and the variation between the AVC and the normal samples, PCA analysis is used. On the basis of the findings of the PCA cluster analysis, it was discovered that there was a statistically significant difference in immune cell infiltration (Figure 9A). We compared the composition of immune cell infiltration in AVC and normal samples using the data matrix of the combined datasets of GSE12644 and GSE51472 (Figure 9C). The study's findings indicated that the fraction of naïve B cells (*P* < 0.05), activated natural killer cells (*P* < 0.05), and monocytes (*P* < 0.05) was significantly higher in normal tissues than in AVC tissues. However, in normal tissues, the ratio of memory B cells (*P* < 0.05), plasma cells (*P* < 0.05), and macrophages M0 (*P* < 0.05) was much lower than in AVC tissues (Figure 9D). Additionally, the relationship between 22 immune cells was analyzed (Figure 9B). Naïve B cells strongly positively connected with activated natural killer cells (r = 0.32), but substantially negatively associated with macrophages M0 (r = −0.31), plasma cells (r = −0.36), and monocytes (r = −0.18). Memory B cells were strongly positively connected with with plasma cells (r = 0.06) and macrophages M0 (r = 0.29), but substantially negatively associated with naïve B cells (r = −0.25), monocytes (r = −0.25), and activated natural killer cells (r = −0.27). Plasma cells were strongly positively connected with memory B cells (r = 0.06) and macrophages M0 (r = 0.05), but substantially negatively associated with naïve B cells (r = −0.36), monocytes (r = −0.01), and activated natural killer cells (r = −0.11). Activated natural killer cells were strongly positively connected with naïve B cells (r = 0.32) and monocytes (r = 0.38), but substantially negatively associated with memory B cells (r = −0.27), macrophages M0 (r = −0.34), and plasma cells (r = −0.11). Monocytes were strongly positively connected with activated natural killer cells (r = 0.38), but substantially negatively associated with naïve B cells (r = −0.18), memory B cells (r =-0.25), plasma cells (r = −0.01), and macrophages M0 (r = −0.35). Macrophages M0 were strongly positively connected with memory B cells (r = 0.29) and plasma cells (r = 0.05), but substantially negatively associated with naïve B cells (r = −0.31), activated natural killer cells (r = −0.34), and monocytes (r = −0.35).

### FN1 and Infiltrating Immune Cells

**Figure 10:**
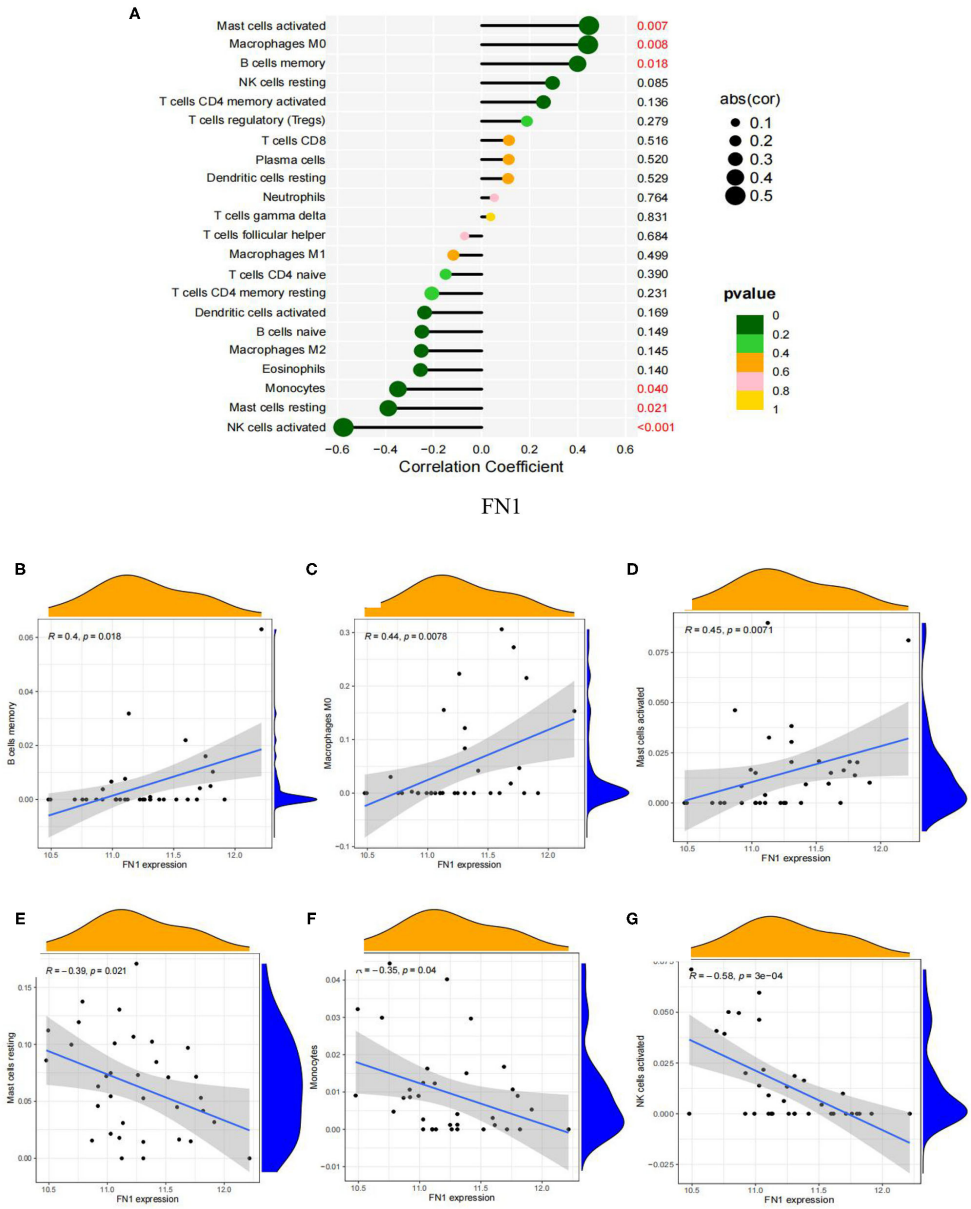
Visualization of the results of immune cell infiltration and FN1 correlation analysis based on the combined data matrix of GSE12644 and GSE51472. **(A)** Correlation between FN1 and infiltrating immune cells. The size of the dots represents the strength of the correlation between genes and immune cells; the larger the dots, the stronger the correlation, and the smaller the dots, the weaker the correlation. The color of the dots represents the *p*-value, the greener the color, the lower the *p*-value, and the yellower the color, the larger the *p*-value < 0.05 was considered statistically significant. **(B)** The correlation analysis in the expression of FN1 and B cells memory. **(C)** The correlation analysis in the expression of FN1 and Macrophages M0. **(D)** The correlation analysis in the expression of FN1 and Mast cells activated. **(E)** Correlation between FN1 and Mast cells resting. **(F)** The correlation analysis in the expression of FN1 and Monocytes. **(G)** The correlation analysis in the expression of FN1 and NK cells activated.

We analyzed the correlation of the above results of immune infiltration with FN1. As shown in Figure 10A, FN1 was positively correlated with memory B cells (r = 0.40, *P* = 0.0018; Figure 10B), macrophages M0 (r = 0.44, *P* = 0.008; Figure 10C) and activated mast cells (r = 0.45, *P* = 0.007; Figure 10D), but significantly negatively correlated with resting mast cells (r =-0.39, *P* = 0.021; Figure 10E), monocytes (r = −0.35, *P* = 0.040; Figure 10F) and activated natural killer cells (r =-0.58, *P* = 0.0003; Figure 10G). The correlation between FN1 and immune cells is presented in Supplementary File 7.

### Identification of Small Molecule Drugs

DEGs were first separated into up-regulated and down-regulated categories, and then enriched with substantially altered genes acquired from treatment with small compounds from the CMap database. As part of the AVC tissue study, yohimbic acid was identified as one of the expected small compounds that might block the expression of genes related with AVC (Table 2).

**Table 2:** List of the 10 most significant small molecule drugs that can reverse the calcified status of AVC.

**AVC tissue**	**CMap name**	**Enrichment**	***P*-value**
	Yohimbic acid	−0.899	0.00192
	PF-00539745-00	−0.769	0.0252
	Hemicholinium	−0.665	0.02847
	Brinzolamide	−0.655	0.03284
AVC vs. normal	Pepstatin	−0.645	0.0377
	Lithocholic acid	−0.612	0.01114
	Minoxidil	−0.567	0.04652
	Salbutamol	0.625	0.02159
	Colchicine	0.634	0.00735
	Physostigmine	0.636	0.04307

*AVC, aortic valve calcification*.

## Discussion

AVC is a term that refers to a collection of aortic valve diseases ranging from calcification to hardness to stenosis (31). Its occurrence rises as a person ages, affecting ~9% of patients over the age of 80 years. Aortic sclerosis is more prevalent, affecting 25-30% of people over the 65 years old and progressing at a rate of around 2% every year to aortic valve stenosis (32). AVC is a complex and dynamic process that is regulated by a variety of physiological and pathological factors such as lipoprotein deposition, inflammatory response, activation of renin-angiotensin-aldosterone, extracellular matrix transformation, and cellular ossification (33, 34), thereby destroying valve-specific cells including endothelial cells as well as interstitial cells, aggravating valvular calcification, causing valve orifice stenosis, and obstructing the left ventricular outflow path (35). For advanced AVC or AVC accompanied by significant clinical symptoms, surgery and transcatheter aortic valve replacement are presently the most effective therapies. However, these procedures are associated with increased costs, risk of mortality, and perioperative and postoperative adverse events including anticoagulation-related bleeding or thrombotic events, and reoperation owing to artificial valve insufficiency (4). Thus, the importance of expanding our understanding of the molecular mechanism of degenerative cardiac valve diseases is emphasized (36, 37). Gene sequencing and bioinformatics technology have advanced substantially in recent decades, making it feasible to evaluate and utilize massive amounts of sequencing data (38). Nevertheless, few studies have examined the association between abnormally expressed genetic markers and immunological invasion in AVC vs. normal tissues. Therefore, through the analysis of AVC and normal tissues, we try to identify potential diagnostic biomarkers and further observe the role of immune cell infiltration in the disease.

To our information, compared to previous studies (39, 40), the analytical approach to filtering the biomarkers of the AVC was first investigated using the lasso and SVM algorithms. Lasso logistic regression, a machine learning method, identifies variables by selecting those with the lowest probability of classification error (41). SVM-RFE is a machine learning technique that has been used in ranking features and selecting the most important features for classification in multiple applications (42). Recently, Akter et al. (43) found that combining multiple machine learning methods could improve predictive performance and thus provide a highly accurate predictive diagnostic model. Thus, taking advantage of the two machine learning methods is beneficial for us to discover potentially important biomarkers, which is very meaningful for our research on AVC. We collected two cohorts from the GEO datasets and analyzed the data in an integrated manner. A total of 337 DEGs were discovered, comprising 203 up-regulated and 134 down-regulated genes. Diseases enriched by DEGs, according to the results of enrichment analyses, were mostly associated with arteriosclerosis and arteriosclerotic cardiovascular disease. Following analysis, it was shown that the enriched pathways were mostly associated with the inflammation and immunological response, such as cytokine-cytokine receptor interactions and chemokine signaling. These results corroborate previous findings that inflammation plays a role in the development and progression of AVC (44, 45) and that AVC is an inflammation-dependent process (46). Initially thought to be a “degenerative” procedure, AVC is now identified as an active condition in which interplay between valvular interstitial cells, the major biological constituent of the aortic valve, and circulatory inflammatory cells and endothelial progenitor cells drive tissue restructuring (47). Numerous findings revealed the presence of many pro-inflammatory cytokines, including tumor necrosis factor, oxidized low-density lipoprotein cholesterol, and interleukin (IL)-1/2/6, in calcified valves (48, 49).

Inflammation is speculated to play a role in the etiology of AVC, with T lymphocytes (50) and macrophages (10) being found in early aortic valve lesions. IL-6 has been reported to perform as a proinflammatory factor, activating biomineralization and osteogenic signaling mechanisms in the cardiovascular system (11, 44, 51, 52). This result is congruent with our findings, validating the credibility of the current study's findings and indicating the critical role of the immune response in AVC. The immunological response is perhaps the most varied and complicated reaction that occurs after the activation of inflammatory processes within the aortic valve, and it has been shown to impact various calcification processes (53). Furthermore, to provide a safe and effective therapy, it is also vital to precisely regulate development of various immune cell types in aortic valve. Thus, novel biomarkers of disorders associated to immune cell infiltration may be discovered using bioinformatics analysis to block related pathways to improve AVC therapy.

Based on two machine-learning algorithms and hub gene identification, one diagnostic marker was identified. Fibronectin is a glycoprotein ligand that is extensively expressed in a variety of cell types that bind cell surfaces and compounds including collagen, fibrin, heparin, DNA, and actin (54). FN1 belongs to this family and plays a role in a variety of biological activities, including cell migration, adhesion, and cytoskeleton structure (55). Aberrant FN1 expression is also associated with a multitude of diseases, including cancer, atherosclerosis, and arthritis (56–58). A previous study showed that osteoblasts are accompanied by type I collagen production during proliferation and differentiation, and found high expression of FN1, implying that osteoblasts are accompanied by upregulation of FN1 during active bone formation (58). Moreover, FN1 is engaged in osteoblast compaction through matrix assembly processes mediated by FN fibrillogenesis cells, which are essential for osteoblast mineralization (59). Yang et al. (60) have recently discovered FN1 as a molecular regulator in promoting osteogenic differentiation *in vitro*. *In vitro*, FN-1 knockdown decreases β-catenin expression and inhibits WNT/-catenin signaling, hindering osteoblast differentiation and mineralization., while overexpression of FN1 stimulates WNT/β-catenin signaling and promotes pre-osteoblast differentiation and mineralization (60). As AVC advances, inflammatory cells inside the subendothelium and fibrosa cause oxidative stress and generate a variety of growth factors and cytokines (11), including transforming growth factors, tumor necrosis factors, interleukin-1b (IL-1b), and receptor activator of nuclear factor-κB ligand. These growth factors and cytokines are major inducers of valve osteoblast development by activating multiple signaling pathways (including Notch, bone morphogenetic proteins and WNT/β-catenin) and establishing an environment conducive to osteogenesis (61). In this study, we believe that by stimulating the WNT/β-catenin signaling pathway, FN1 may play a critical role in the development of osteogenesis in calcified aortic valve disease. In addition, Jun et al. (62) revealed that the expression and release of Fn had a substantial impact on the effects of ATP and caspase-1 generated by inflammasome activators. Moreover, As an endogenous danger signal, it boosts the inflammatory process by stimulating caspase-1 and resulting in the death of inflammatory cells. The high concentration of FN-fibrin complex in plasma can promote the release of inflammatory factors from tissues to the circulation during the progression of a variety of diseases, including thrombosis, plaque formation, the development and progression of atherosclerosis, and aging arteries Wall lesions, which further aggravates the inflammatory response and promotes disease progression (63). FN-fibrin complexes have been associated with numerous diseases, including diabetes mellitus (64), rheumatoid arthritis (65), osteoarthritis (66), chronic obstructive pulmonary disease (67), and coronary artery disease (68). Thus, our findings suggest that the FN1 may be critical in the evolution of the inflammatory process in calcified aortic valve disease.

By using CIBERSORT to analyze the immune cell types between AVC and normal samples, it is found that the basic biological processes related to AVC are closely related to a variety of immune cells. The analysis results found that memory B cells and macrophages M0 are significantly expressed in AVC tissues, and naïve B cells, plasma cells, activated natural killer cells, and monocytes are significantly expressed in normal tissues. In addition, FN1 was significantly expressed in the AVC tissue. Correlation analysis showed that memory B cells, macrophages M0, activated mast cells were positively correlated with FN1, and resting mast cells, monocytes, as well as activated natural killer cells were negatively correlated with FN1, which meaning that the high expression of FN1 tissues greatly promotes the infiltration of memory B cells, macrophages M0 to the tissues, on the contrary, may inhibit the activation of activated natural killer cells and monocytes. Therefore, FN1 and a variety of inflammatory cells participate in the progress of AVC, which provides effective evidence for further research on the molecular mechanism of AVC in the future. Inflammatory and immune circulating cells, such as memory B cells, neutrophils and macrophages, have been shown to have a significant influence in the development of heart-related diseases in the prior (69), which is consistent with our research. Endothelial damage caused by increased mechanical stress and decreased shear stress is thought to be the first stage in AVC development (70). As a consequence, localized subendothelial plaque-like lesions may be detected, involving infiltration of inflammatory cells, buildup of subendothelial lipids, breakdown of the extracellular matrix, and fracturing of the surrounding elastic lamina (71). Furthermore, oxidized lipids also induce an inflammatory response in valve tissue. In response to the uptake of oxidized lipids, macrophages, monocytes, CD4+ and CD8+ T lymphocytes, and mast cells in the surrounding area become activated (10). Monocytes and macrophages stimulate the osteogenic differentiation of valvular interstitial cells and calcification via secretion of tumor necrosis factor, which is followed by activation of NF-κB and IL-1β and IL-6 (72). Following this, as a result of endothelial dysfunction and inflammation, localized cell death leads to the release of apoptotic bodies, which promote microcalcification. This process is further promoted by the release of extracellular vesicles by macrophages and valvular interstitial cells (73, 74). Such calcification induces an even stronger immune response, resulting in a vicious loop and ultimately leading to the disease's propagation phase. AVC patients' natural killer cells accumulate in cardiac valves and blood circulation, which has been associated to an increase in their valve pressure gradient, according to Mazur's research (75). We suggest that the enormous amount of evidence described earlier, combined with our present study findings, indicates that a variety of infiltrating immune cells play an essential role in AVC and future study should be focused on this point.

The CMAP database, which contains 7,056 gene expression profiles triggered by 1,309 small compounds, is commonly used to investigate the unknown functions of currently available medications in disease (76). To begin, the 337 DEGs were categorized into up- and down-regulated categories. Then, these genes from two groups were uploaded to the CMAP database to identify possible small therapeutic compounds, with a cut-off value of *P* < 0.05 used. Finally, enrichment scores (−1 to + 1) were generated to determine the similarity between genes and medicines. Specifically, an enrichment score > 0 indicated that the molecules had potential synergistic effects with AVC, implying that they could mimic the biological state of the cells that induce AVC; whereas, an enrichment score < 0 indicated that the molecules had potential inhibitory action with AVC, implying that they could reverse the AVC state and serve as therapeutic agents. We therefore successfully identified the top 10 small molecules with the smallest enrichment scores, of which yohimbic acid was the first. However, very little research has been done on yohimbic acid, with only a few studies reporting its vasodilatory (77) and sympathetic activity effects (78). There is still a lack of a large pharmacological base to verify its function. Therefore, further studies are necessary to confirm its reversal or delaying effect on AVC in the future.

This study has several limitations. First, it was retrospective in nature; thus, there was a lack of critical clinical information. Secondly, the GSE83453 validation cohort included a small number of cases, which could be considered as a constraint. Thirdly, it is challenging to account for critical aspects such as area, race, and age. Considering that AVC is caused by a multitude of environmental and genetic variables, certain unmeasurable factors, such as geography and family history, need additional investigation. Additionally, potential key genes should be validated in clinical samples using RT-qPCR. However, since there are not enough normal aortic valve samples in our department, it is difficult to carry out the verification experiment. In the future, we will collect the aortic valve tissue transplanted in our department to further explore the mechanism of FN1 on AVC. Finally, the method by which these genes operate is unknown. Additional evidence is evaluated to confirm the biological basis.

In conclusion, we present bioinformatic evidence demonstrating that FN1 might be a potential biomarker for discrimination of AVC. Yohimbic acid was also identified as a potential anti-calcification drug in calcified aortic disease (*P* < 0.05). Given that the pathogenic processes of AVC remain unknown, our findings may have a wide influence on AVC biology and treatment. Nonetheless, larger sample sizes and further mechanistic investigations are required to corroborate our conclusions.

## Data Availability Statement

The datasets presented in this study can be found in online repositories. The names of the repository/repositories and accession number(s) can be found in the article/Supplementary Material.

## Author Contributions

TX was engaged for conception and writing—original draft production. XZ and SH for methodology. LP, T-CZ, TF, and Y-HD for sofware. Y-XL for writing—review and editing. All authors have reviewed and approved the published version of the work.

## Funding

This study was sponsored by funds from the Chinese National Natural Science Foundation (No. 82060093), the Science and Technology Department of Yunnan Province (No. 202101AY070001-211 and No. 2019FE001-268), and Yunnan Province's Key Laboratory of Cardiovascular Disease (No. 2018DG008).

## Conflict of Interest

The authors declare that the research was conducted in the absence of any commercial or financial relationships that could be construed as a potential conflict of interest.

## Publisher's Note

All claims expressed in this article are solely those of the authors and do not necessarily represent those of their affiliated organizations, or those of the publisher, the editors and the reviewers. Any product that may be evaluated in this article, or claim that may be made by its manufacturer, is not guaranteed or endorsed by the publisher.

## Abbreviations

AVC: aortic valve calcification
DEGs: differentially expressed genes
PPIN: protein–protein interaction network
FN1: fibronectin 1
GO: Gene Ontology
DO: Disease Ontology
GSEA: Gene set enrichment analysis
STRING: The Search Tool for the Retrieval of Interacting Genes
PPI: protein–protein interaction
LASSO: least absolute shrinkage and selection operator
SVM-RFE: support vector machine-recursive feature elimination
KEGG: Kyoto Encyclopedia of Genes and Genomes
CXCL8: C-X-C motif chemokine ligand 8
CXCR4: C-X-C motif chemokine receptor 4
CCR5: C-C motif chemokine receptor 5
MMP9: matrix metallopeptidase 9
SYK: spleen-associated tyrosine kinase
TYROBP: transmembrane immune signaling adaptor
CXCL12: C-X-C motif chemokine ligand 12
SDC1: syndecan 1
TLR2: toll-like receptor 2
IL: interleukin
NF: necrosis factor.
